# Psychosocial and clinical characteristics in Takotsubo syndrome

**DOI:** 10.1186/s13293-025-00729-0

**Published:** 2025-06-16

**Authors:** Okezi Obrutu, Yujie Cui, Jenna Maughan, Paul Marano, Janet Wei, Martha Gulati, Marie Lauzon, Romana Herscovici, Chrisandra Shufelt, Natalie Rojas, Benita Tjoe, Thomas Rutledge, C. Noel Bairey Merz

**Affiliations:** 1 Barbra Streisand Women’s Heart Center, Cedars-Sinai Smidt Heart Institute, 127 S. San Vicente Blvd AHSP Suite A3206, Los Angeles, CA 90048 USA; 2 Biostatistics and Bioinformatics Research Center, Cedars-Sinai Medical Center, Los Angeles, CA USA; 3 Smidt Heart Institute, Cedars-Sinai Medical Center, Los Angeles, CA USA; 4 The Lev Leviev Heart Center, Chaim Sheba Medical Center, Tel-Hashomer, Israel; 5 Women’s Health Research Center, Mayo Clinic, Jacksonville, FL USA; 6 Queen’s Medical Center, Honolulu, HI USA; 7 VA San Diego Healthcare System, San Diego, CA USA; 8 Department of Psychiatry, University of California, San Diego, CA USA

**Keywords:** Takotsubo syndrome, PTSD, Depression, Psychosocial characteristics, Stress cardiomyopathy

## Abstract

**Background:**

Takotsubo Syndrome (TTS) is an acute form of heart failure that disproportionately impacts post-menopausal women. The brain-heart connection is considered a pathway for TTS pathophysiology leading to investigations of the role of psychological, psychosocial, and personality factors in TTS.

**Objectives:**

We compare psychosocial characteristics among a subset of individuals with confirmed TTS and those who had symptoms adjudicated as non-TTS in our online Takotsubo registry (*n* = 104). We also evaluate differences in TTS clinical characteristics among those with and without symptoms of PTSD and depression.

**Methods:**

The Smidt Heart Institute Takotsubo registry enrolls individuals with a history of TTS sourced through physician referrals, medical records review, peer- and self-referrals. Psychosocial characteristics were assessed using questionnaires validated in acute coronary syndrome populations. Hedge’s g effect sizes were computed to compare differences in perceived stress, depression symptoms, and post-traumatic stress disorder (PTSD) symptoms relative to TTS status.

**Results:**

Compared to participants confirmed to be non-TTS, those with adjudication-confirmed TTS had worse mean psychosocial scores (indicative of worse psychosocial characteristics). After adjusting for age at event, BMI, race, and smoking status, the Hedge’s g effect size for depressive symptoms was moderate [0.60 (-0.03, 1.22)] while effect sizes for other psychosocial measures were minimal (Trait anxiety: [0.01 (-0.58, 0.60)], PTSD symptoms [0.13 (-0.46, 0.73)], perceived stress [0.06 (-0.53, 0.65)]. Effect sizes were relatively lower following adjustment, largely driven by participants’ age at first event. Individuals with elevated PTSD symptoms were significantly younger at their first TTS event compared to those with minimal or no symptoms (54 ± 8 vs. 61 ± 10; *p* = 0.005). QTc was relatively longer among individuals with elevated PTSD symptoms (483 ± 40 msec vs. 465 ± 32 msec; *p* = 0.08) and elevated depressive symptoms (481 ± 33 msec vs. 464 ± 36 msec; *p* = 0.07), although the differences were not statistically significant.

**Conclusions:**

Psychosocial characteristics including PTSD, depression, and stress are common among women with TTS, and age at the time of TTS event is a potentially important moderator of this relationship. We did not find Trait-anxiety or early childhood trauma to be associated with TTS in our cohort.

**Supplementary Information:**

The online version contains supplementary material available at 10.1186/s13293-025-00729-0.

## Abstract (plain language summary)

Takotsubo syndrome (TTS) is a form of heart failure that is linked to emotional and physical stressors. It commonly affects women after menopause. Literature suggests that the brain and heart are connected in this illness, so we studied how stress, depression, and other psychological factors might play a role. This study compared people with TTS to those with similar symptoms who didn’t have TTS. We used surveys to measure stress, depression, Post-Traumatic Stress Disorder (PTSD), and other psychosocial characteristics when participants enrolled into the study. Clinical characteristics were determined from a retrospective review of participants’ medical records during their TTS event.

We found that individuals with confirmed TTS had scores that were representative of higher levels of depression, PTSD, and perceived stress compared to the group without TTS. Younger people with TTS were more likely to have PTSD symptoms. On average, people with PTSD symptoms had their first TTS event at age 54, while others had it at age 61. Depression and PTSD seemed to have a more considerable effect on TTS than anxiety or early-life trauma. The study shows that feelings like stress, depression, and PTSD are common in people with TTS, and age might make a difference in how these feelings affect the heart. Our findings offer additional insight into psychosocial features and associated clinical characteristics in TTS.

**Supplementary Information:**

The online version contains supplementary material available at 10.1186/s13293-025-00729-0.

## Highlights

Participants with confirmed TTS had a higher burden of adverse psychosocial characteristics including PTSD, depression, and stress compared to those with non-TTS.

There were no significant differences in age, BMI, smoking history, menopausal status, and medical history between those with confirmed TTS and non-TTS.

PTSD was associated with younger age at first event among individuals with confirmed TTS. Additionally, as a covariate, age was negatively correlated with perceived stress, depression, and PTSD symptom severity scores.

QTc was relatively longer among participants with elevated depression and PTSD symptoms.

**Supplementary Information:**

The online version contains supplementary material available at 10.1186/s13293-025-00729-0.

## Introduction

Takotsubo Syndrome (TTS) is an acute form of heart failure linked to emotional and physical stressors. TTS affects 2% of individuals presenting with suspected acute coronary syndrome (ACS) [1]. Post-menopausal women are more often affected, and the prevalence of TTS in individuals presenting with symptoms of ACS increases to 10% when only women are considered. An analysis of the United States (US) National Inpatient Sample dataset showed that 90% of individuals with TTS were ≥ 50 years of age, and 90% were female [1, 2].

Although the etiology of TTS is not fully understood, the brain-heart connection is considered as a pathway for its pathophysiology [3, 4]. The brain-heart hypothesis suggests that stress activates the sympathetic nervous system leading to excessive release of catecholamines and toxic effects on the heart [4]. This has led to investigations of the role of psychological, psychosocial, and personality factors in TTS. For example, studies have shown higher prevalence of anxiety and post-traumatic stress disorder (PTSD) in TTS patients compared to those with ACS [5]. However, current evidence about the role of depression in TTS is inconsistent, with some studies showing a higher prevalence compared to the general population and others finding no relationship [6].

### Study aims

We evaluated self-reported psychosocial and clinical characteristics among individuals with confirmed prior TTS and compared them to those who had ACS symptoms adjudicated as non-TTS based on the International Takotsubo (InterTAK) Diagnostic Criteria in an online TTS registry.

## Methods

The Smidt Heart Institute Takotsubo Registry is an online retrospective registry of individuals with a history of TTS. Details about the registry design, including adjudication methodology, have been previously published [7]. At enrollment, participants are requested to fill detailed psychosocial questionnaires in addition to medical history questionnaires. The duration between the first acute event and enrollment was a median of 1 year. Adjudication is based on review of detailed medical records and investigation results, including troponin levels & ECGs at time of the event, echocardiograms/ventriculograms, coronary angiograms, and hospitalization summaries [7]. The 2018 InterTAK criteria is used to determine TTS or non-TTS status based on review findings [8]. Subsequent events are identified based on participant reports in annual follow up surveys. The adjudication process is repeated for any reported event. Our current analysis was limited to individuals in the registry who completed psychosocial questions and had completed adjudication data for ≥ 1 TTS event (*n* = 104). This includes participants who were adjudicated as confirmed TTS and non-TTS. No male participants met the criteria for this analysis. Individuals in the non-TTS group likely presented with Takotsubo-like symptoms and were diagnosed as TTS at discharge, however, our thorough adjudication based on their medical records determined that their events were non-TTS. We have included them as a comparison group in this analysis because they are similar in terms of their presentation, yet different in terms of their adjudicated diagnosis. Other TTS studies have also used similar populations with cardiovascular disease (CVD) as comparison groups to highlight distinctions between TTS and these conditions [9, 10].

### Measures

**Fig. 1:**
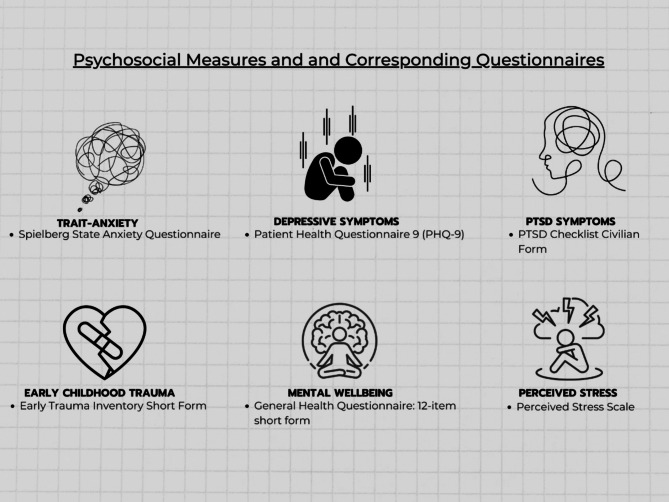
Psychosocial questionnaires mapping

Psychosocial characteristics were assessed using questionnaires validated in ACS populations (Fig. 1) [11, 12]. Anxiety symptoms were assessed based on the Spielberger State-Trait Anxiety Inventory (STAI) Form Y-2, stress levels were assessed using the Post-Traumatic Stress Disorder (PTSD) Checklist Civilian Form (PCL-C) and Perceived Stress Scale (PSS) questionnaires. In addition, depression symptoms were assessed using the Patient Health Questionnaire 9 (PHQ-9), and history of trauma was determined from the Early Trauma Inventory Self Report – Short Form (ETISR-SF). The mental component summary score (MCS) of the 12-Item Short Form Survey (SF-12) was used to determine mental wellbeing. Higher scores represent worse psychosocial status for all characteristics except the SF-12 MCS. Data on clinical characteristics at TTS events, including electrocardiogram and echocardiogram features, were collated by adjudicating clinicians from participant medical records.

### Data analysis

Patient demographic, clinical, and psychosocial characteristics and scores were summarized using means and standard deviations for continuous variables, and frequencies and percentages for categorical variables. Welch two sample t-test (for continuous variables) and Pearson’s Chi square test or Fisher’s exact test (for categorical variables) were used to compare the differences in demographic and clinical characteristics. Hedge’s g effect sizes were computed to assess the extent of the differences in psychosocial characteristics (Trait-anxiety, perceived stress, depression symptoms, PTSD symptoms, early childhood trauma, mental wellbeing scores) relative to TTS status, correcting for bias in small samples. This metric represents the mean difference between two groups relative to the pooled standard deviation, with values of 0.2, 0.5, and 0.8 typically indicating small, medium, and large effects, respectively. These Hedge’s g calculations were performed both with and without adjustments for variables such as age at first event, race, body mass index (BMI), and smoking status, due to clinical rather than statistical relevance. Based on published cut-offs for clinically significant symptom levels on the PCL-C and PHQ-9 [13, 14], we determined participants’ PTSD and depression symptom severity as either normal or elevated. Among those with confirmed TTS, we used Wilcoxon rank sum test, Pearson’s Chi square or Fisher’s exact test to examine the association between TTS clinical characteristics and PTSD/depression. The significance level for two-tailed hypothesis tests was set at 0.05. All statistical analyses were conducted using statistical software R (R Core Team, v4.2.1).

## Results

Participant demographic characteristics are summarized in Table 1. A total of 104 women were included in this analysis, 88 (85%) of which had adjudication-confirmed TTS. There were no significant differences in age, BMI, smoking history, menopausal status, and medical history between those with confirmed TTS and non-TTS (Table 1).

**Table 1:** Characteristics of women with and without Takotsubo syndrome

	Frequency (%)		
	**Confirmed TTS** **(** ***n*** **= 88)**	**Non-TTS** **(** ***n*** **= 16)**	**p-value** †
Age at study enrolment (years)*	62.1 ± 10.4	61.3 ± 10.5	0.80
**Race (%)**			0.09
White	81 (92.1)	12 (75.0)	
Other	7 (7.9)	4 (25.0)	
**Hispanic Ethnicity (%)**	4 (4.6)	3 (18.8)	0.07
**BMI (kg/m**^**2**^)*	25.4 ± 6.3	27.6 ± 7.3	0.30
**Post-Menopausal (%)**	75 (88.2)	15 (93.8)	0.99
**History of Smoking (%)**	33 (38.4)	5 (31.3)	0.78
**History of Hypertension (%)**	37 (43.0)	6 (37.5)	0.87
**History of Diabetes (%)**	8 (9.3)	1 (6.3)	0.99
**History of Dyslipidemia (%)**	30 (34.9)	6 (37.7)	0.99
**Family History of Coronary Artery Disease (%)**	40 (46.5)	11 (68.8)	0.30

*Mean ± SD

† No group differences between women with and without TTS

TTS = Takotsubo Syndrome; BMI = Body Mass Index

### Psychosocial characteristics

**Fig. 2:**
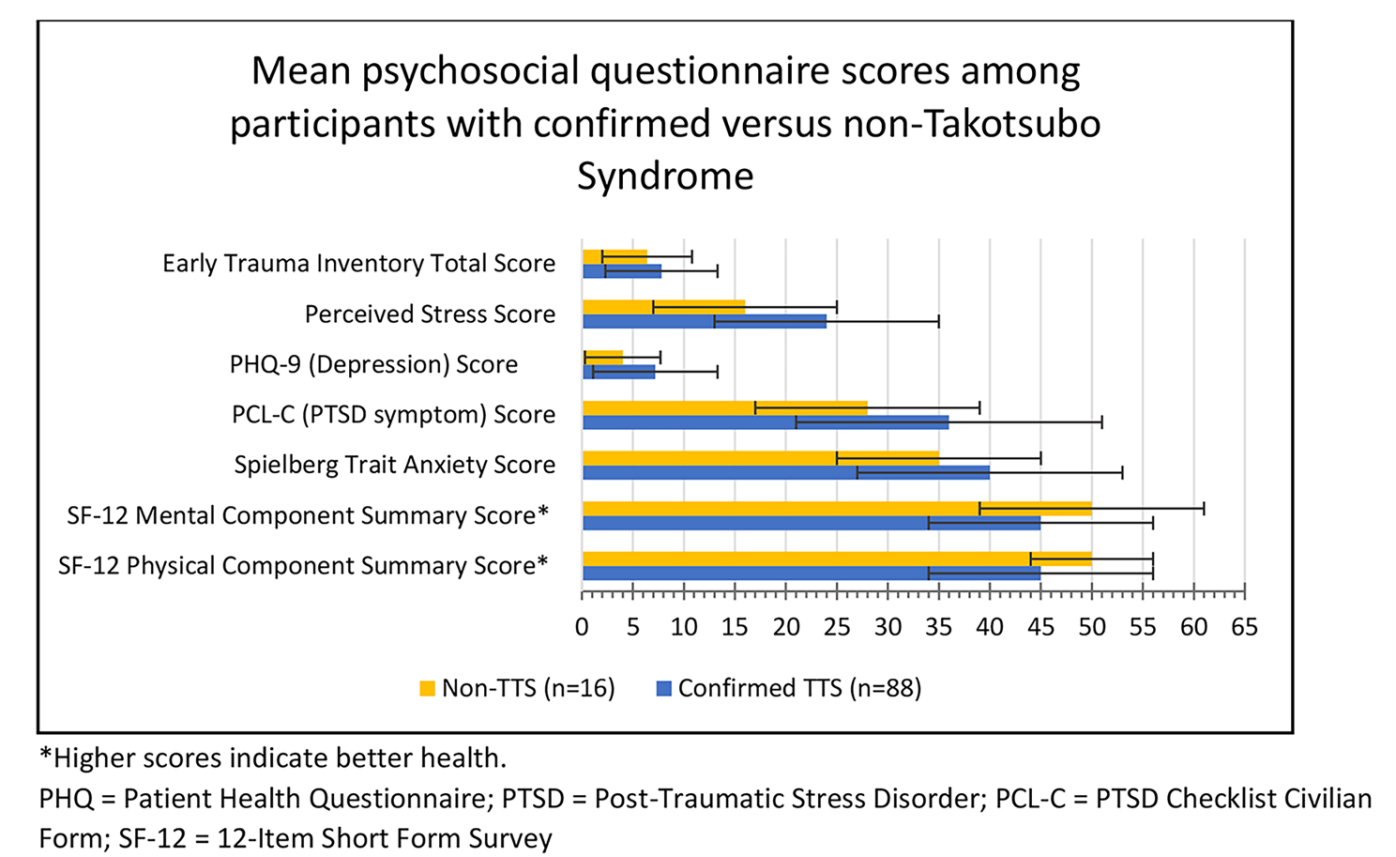
Mean psychosocial questionnaire scores

Compared to participants with non-TTS, the group with confirmed TTS had worse mean psychosocial scores (Fig. 2). Crude analyses showed moderate Hedge’s g effect sizes when comparing scores for Trait Anxiety [0.39 (95% CI: -0.15, 0.92)], PTSD symptoms [0.55 (95% CI: 0.00, 1.10)], depression symptoms [0.54 (95% CI: -0.02, 1.09)], perceived stress [0.71 (95% CI: 0.16, 1.25)], and SF-12 MCS [-0.49 (95% CI: -1.03, 0.04)]. These effect sizes range from small to medium by conventional standards and were statistically significant for PTSD symptom severity and perceived stress, however, the effect sizes reduced after adjustment for age at event, BMI, race, and smoking history, and were no longer statistically significant (Table 2). The reduction in effect sizes was largely driven by participants’ age at first reported event.

**Table 2:** Prevalence of adverse psychosocial characteristics

Psychosocial characteristics*	TTS Status		Crude		Adjusted †	
	Confirmed TTS (*n* = 88)	Non-TTS (*n* = 16)	Hedges’ g Effect size	95% CI	Hedges’ g effect size†	95% CI
**Trait Anxiety**	40 ± 13	35 ± 10	0.39	-0.15, 0.92	0.01	-0.58, 0.60
**PCL-C (PTSD symptom) scores**	36 ± 15	28 ± 11	**0.55**	0.00, 1.10	0.13	-0.46, 0.73
**n (%) with PTSD**	24 (30%)	2 (13%)	--		--	
**PHQ-9 (Depression) scores**	7.2 ± 6.1	4.0 ± 3.7	**0.54**	-0.02, 1.09	**0.60**	-0.03, 1.22
**n (%) with clinical depression**	21 (27%)	1 (6.7%)	--		--	
**Perceived Stress Scores**	24 ± 11	16 ± 9	**0.71**	0.16, 1.25	0.06	-0.53, 0.65
**ETI Total Score**	7.8 ± 5.5	6.4 ± 4.4	0.26	-0.29, 0.81	0.11	-0.48, 0.70
**Early Trauma Inventory**						
Significant history of General Trauma	40 (49%)	4 (27%)	--		--	
Significant history of Physical Abuse	15 (19%)	1 (6.7%)	--		--	
Significant history of Emotional Abuse	31 (38%)	6 (40%)	--		--	
Significant history of Sexual Abuse	40 (49%)	6 (40%)	--		--	
Significant history of Any Childhood Trauma	44 (54%)	6 (40%)	--		--	
**12-Item Short Form Survey (SF-12)**						
Physical Component Summary Score (PCS)	45 ± 11	50 ± 6	-0.46	-0.99, 0.08	**-0.78**	-1.38, -0.18
Mental Component Summary Score (MCS)	45 ± 11	50 ± 11	-0.49	-1.03, 0.04	0.01	-0.58, 0.60
**n (%) with PTSD and Depression**	13 (15%)	1 (6.3%)	--		--	

PHQ-9 = Patient Health Questionnaire 9; PTSD = Post-Traumatic Stress Disorder; PCL-C = PTSD Checklist Civilian Form

† Effect sizes after adjusting for age at event, BMI, race, and smoking status

*Except for SF-12, higher scores indicate worse psychosocial status. Lower SF-12 scores indicate worse health

Medium effect sizes (g ≥ 0.5) in **bold**

When included as a covariate, age at first event showed a negative correlation with perceived stress, depression, and PTSD symptom severity scores. Specifically, PCL-C total score exhibited a more substantial decrease of 0.37 with each year increase in age at first event, and this association was found to be statistically significant (*p* = 0.01). PSS score decreased by 0.18 and PHQ-9 score decreased by 0.08 for each incremental year in age at first event, although these correlations did not reach statistical significance, (*p* = 0.10 and 0.20 respectively).

### Demographic and clinical characteristics associated with PTSD and depression among those with confirmed TTS

About 15% of individuals with confirmed TTS had both elevated PTSD and depression symptoms, compared to 6.3% of those with non-TTS. Participants with confirmed TTS and a positive history of PTSD were younger at their first TTS event compared to those with minimal/no PTSD [Mean ± SD: 54 ± 8 vs. 61 ± 10, *p* = 0.005] (Table 3). They also had longer QTc intervals compared to those with minimal/no PTSD symptoms [Mean ± SD: 483 ± 40 msec vs. 465 ± 32 msec; *p* = 0.08 (Table 3). Additionally, confirmed TTS participants with elevated depression symptoms had a trend toward longer QTc intervals compared to those with normal depression levels [Mean ± SD: 481 ± 33 msec vs. 464 ± 36 msec; *p* = 0.07 (Table 4). There were no other significant differences in demographic and TTS characteristics including echocardiogram and angiogram features between both groups (Table 4).

**Table 3:** Association between PTSD symptom status* and demographic/clinical characteristics in participants with confirmed TTS

	Elevated PTSD symptoms *n* = 24	Minimal/No PTSD symptoms *n* = 57	*p*-value
**Age (years)**†	58 ± 9	64 ± 10	**0.02**
**BMI (kg/m**^**2**^**)**†	26.9 ± 7.2	25.3 ± 5.7	0.40
**Age at first TTS event**†	54 ± 8	61 ± 10	**0.005**
**Takotsubo Trigger (Self-report)**	17 (71%)	38 (67%)	0.70
**Self-reported trigger type**			0.20
Emotional	10 (59%)	11 (29%)	
Physical	3 (18%)	12 (32%)	
Both emotional and physical	4 (24%)	14 (37%)	
Unknown	0 (0%)	1 (2.6%)	
**Takotsubo Trigger (Adjudicated)**	20 (83%)	38 (68%)	0.20
**Adjudicated trigger type**			0.60
Emotional	7 (35%)	17 (45%)	
Physical	10 (50%)	18 (47%)	
Both emotional and physical	3 (15%)	3 (7.9%)	
**Total TTS events**			0.04
1	19 (79%)	48 (84%)	
2	1 (4.2%)	8 (14%)	
3	2 (8.3%)	1 (1.8%)	
4	2 (8.3%)	0 (0%)	
**Electrocardiogram at first TTS event**			
Inferior ST segment elevation	0 (0%)	6 (11%)	0.20
Anterior ST segment elevation	4 (17%)	9 (16%)	> 0.9
Lateral ST segment elevation	6 (25%)	9 (16%)	0.40
QTc (msec)†	483 ± 40	465 ± 32	0.08

*Based on PCL-C scores

†Mean ± SD

**Table 4:** Association between PHQ-9 based depression diagnosis and demographic/clinical characteristics in participants with confirmed TTS

	Depression *n* = 21	No Depression *n* = 56	*p*-value
**Age**†	60 ± 11	63 ± 10	0.40
**BMI**†	25.4 ± 7.1	25.8 ± 5.8	0.80
**Age at first TTS event**†	57 ± 10	59 ± 10	0.30
**Takotsubo Trigger (Self-report)**	14 (67%)	41 (73%)	0.60
**Self-reported trigger type**			0.15
Emotional	6 (43%)	15 (37%)	
Physical	1 (7.1%)	14 (34%)	
Both emotional and physical	7 (50%)	11 (27%)	
Unknown	0 (0%)	1 (2.4%)	
**Takotsubo Trigger (Adjudicated)**	16 (80%)	39 (70%)	0.40
**Adjudicated trigger type**			0.50
Emotional	6 (38%)	16 (41%)	
Physical	7 (44%)	20 (51%)	
Both emotional and physical	3 (19%)	3 (7.7%)	
**Total TTS events**			> 0.90
1	18 (86%)	45 (80%)	
2	2 (9.5%)	7 (13%)	
3	7 (50%)	11 (27%)	
4	0 (0%)	1 (2.4%)	
**Electrocardiogram at first TTS event**			
Inferior ST segment elevation	0 (0%)	6 (11%)	0.20
Anterior ST segment elevation	4 (19%)	9 (16%)	0.70
Lateral ST segment elevation	3 (14%)	11 (20%)	0.70
QTc (msec)†	481 ± 33	464 ± 36	0.07

† Mean ± SD

## Discussion

In this study comparing participants in an on-line registry with confirmed TTS versus those adjudicated to have a non-TTS ACS, we found a high burden of adverse psychosocial characteristics in participants with confirmed TTS. Scores based on validated questionnaires reflected worse psychosocial characteristics in the confirmed TTS participants; however, there was no statistically significant difference after adjustment for clinically relevant covariates. Notably, we found a higher prevalence of PTSD – defined by elevated PCL-C scores – among individuals with confirmed TTS (30%) compared to the general population (Lifetime prevalence – 6% in general and 10–12% in females) [15, 16]. Our participants also had a higher prevalence than the reported 11–22% among women veterans [17, 18]. Consistent with our findings, another study showed higher prevalence of PTSD symptoms in patients with TTS versus MI post-discharge and healthy controls (β = 0.55, *p* < 0.05; β = 0.92, *p* < 0.01 respectively) [5].

Additionally, the prevalence of depression symptoms among our participants with confirmed TTS was higher than the age-standardized prevalence among US adults from the 2020 Behavioral Risk Factor Surveillance System (BRFSS) data (27% vs. 18.5%) [19]. A prior study in a cohort of 27 patients similarly showed that individuals in the acute phase of TTS experienced a relatively high prevalence of depression (26%) [20]. Nonetheless, the PHQ-9 was used to assess depression in that study during the acute hospitalization phase, compared to our study where the assessment was done at registry enrollment (a median of 1-year after the first event). The similar degree of depression in both cohorts suggests that depressive symptoms in patients who develop TTS may be persistent rather than temporal. This contrasts with a prior study that did not find depression to be different in TTS when compared with ACS controls (mean HADS depression score = 4.3 ± 3.7 for TTS cases vs. 4.0 ± 3.1 for ACS controls; *p* = 0.61) [10].

Although the prevalence rates of PTSD and depression were comparatively higher in the TTS versus non-TTS group in our study (30% vs. 13%; *p* = 0.33 and 27% vs. 6.7%; *p* = 0.11), these differences were not statistically significant. This lack of statistical significance may be attributed to the smaller sample size in one of the groups, which reduced our statistical power to detect differences. Notably, only 15% of patients with confirmed TTS had both PTSD and depression, suggesting that there may be different pathways and associations between these conditions and TTS.

In contrast with studies reporting higher rates of anxiety among individuals with TTS compared to control groups [5, 10, 21], we did not find significant differences in Trait-anxiety scores between our confirmed TTS and non-TTS groups. Individuals with ACS and healthy volunteers were distinct control groups in the referenced studies. The conflicting results may be due to the different tools used to assess anxiety. For instance, our study utilized the STAI to assess anxiety, while the study by Goh et al. utilized the Hospital Anxiety and Depression scale (HADS). Other tools that were used for anxiety include the anxiety subscale of Symptoms Checklist (SCL-90), 8-item anxiety subscale of Hopkin’s Symptoms Checklist (ASS) and history of anxiety based on medical records [22]. Additionally, our comparison group was likely different from other studies due to their presentation with TTS-like symptoms and with a likely diagnosis of myocardial infarction with non-obstructive coronary arteries (MINOCA) or ACS presentation, making this comparison population different from the general population. Furthermore, these participants likely believed or were told that they had TTS, which could have affected how they responded to the psychosocial questionnaires in this study or otherwise reported their emotional and stress symptoms.

The mean PSS score, representing perceived stress levels, among participants with confirmed TTS in our study was 24 ± 11. This score is lower than previously reported scores among individuals at the onset of acute TTS (median = 30.5) but higher than those at 12-month follow-up (median = 20.5) [23]. Qualitative interviews by Sundelin et al. confirmed the presence of long-term stress around the period when participants developed TTS, suggesting that their PSS scores may have been higher if measured quantitatively at that time. Higher levels of perceived stress among TTS participants are supported by work by Salmoriago-Blotcher et al., who demonstrated a higher PSS score of 14.6 ± 7.91 one-month post-TTS compared to patients with myocardial infarction 11.51 ± 9.89 and healthy controls 9.08 ± 8.07 [5].

History of early childhood trauma was comparable between the TTS and non-TTS groups in our study (54% vs. 40%), in congruence with a prior study that showed similar levels of childhood trauma between individuals with TTS and myocardial infarction [9]. Based on 2011–2020 BRFSS data, Swedo et al. have reported a prevalence of ≥ 1 adverse childhood experiences (ACEs) in 70% of US adults and ≥ 4 in 17% of US adults [24]. However, the instruments for measuring childhood trauma (or ACEs) across these studies were different, so the results are not directly comparable. Additionally, the similarities in medical history between our TTS and non-TTS group may explain our finding of no significant differences in childhood trauma history.

Younger age at first event was significantly associated with higher PTSD symptom scores in our study. There was also a pattern of higher PSS and depression scores with younger age at first event (although this was not statistically significant). This relationship between age, psychosocial scores, and TTS indicates that psychosocial factors likely play a stronger role in TTS pathophysiology among younger women, however, future research is necessary to confirm the nature and direction of this relationship. Our hypothesis is that there are differences in underlying predispositions among various age groups – perhaps in younger women, PTSD symptoms tend to create the necessary background for TTS, while in older women, the hormonal changes of menopause are the more common predisposing factor. Screening middle-aged women for adverse psychosocial characteristics at routine clinic visits could be considered as a possible pathway to delay onset of TTS if similar findings are replicated in future research. There is evidence in literature for a different clinical profile among pediatric TTS populations [25], including a higher prevalence of physical triggers and higher rates of cardiogenic shock. Although our registry does not enroll children, this finding offers support for evaluating distinctions in risk factors, presentation, and outcomes among participants based on their age at first event.

We adjusted for age at first event, race, BMI, and smoking status in assessing the differences in psychosocial scores between the TTS and non-TTS groups. This was based on the established association between these factors and CVD risk from a clinical perspective. A different study [26] found that 17% of individuals with TTS were current smokers, which is lower than the 38% of our TTS group with a history of smoking. Our figures are likely higher because we assessed any history of smoking. In the referenced study, smoking was associated with longer in-hospital stays in TTS but had no independent impact on in-hospital complications or long-term mortality. We did not assess the relationship between smoking and TTS clinical features in this analysis. There is mixed data on the association between race [27] and TTS prognosis, with several studies indicating no difference between races. However, one study reported more extended hospital stays among African Americans compared to Whites, and another study found differences in mortality among patients of different races with secondary TTS. A nonlinear relationship has been reported between BMI and TTS in-hospital mortality [28]: individuals who were underweight had higher mortality compared to individuals with normal weight, while obesity was not significantly associated. Another study found that significantly higher annual, 3-year, and 5-year mortality rates were in the group with BMI < 18.5 kg/m^2^, while overweight patients (25 ≥ BMI < 30 kg/m^2^) had the best prognosis [29].

The group with clinically elevated PTSD symptoms self-reported relatively higher levels of emotional triggers (59% vs. 29%), while those with clinically elevated depression symptoms reported relatively higher levels of both emotional and physical triggers (50% vs. 27%); although these differences were not statistically significant. However, trigger types based on self-report varied widely from what was determined from medical records (emotional triggers in PTSD vs. no PTSD: 35% vs. 45%; both emotional and physical triggers in depression vs. no depression: 38% vs. 41%). Our analysis did not find PTSD or depression symptoms to be significantly associated with TTS recurrence. Based on a systematic review, Oliveri et al. determined that pre-admission psychiatric disorders were associated with a higher risk of TTS recurrence [6]. We are unable to replicate their analysis because our retrospective study design did not conduct diagnostic interviews for mental health conditions and did not collect data on psychiatric conditions that pre-dated TTS events.

Confirmed TTS participants with clinically elevated depression symptoms had relatively higher QTc (msec) than those with no depression symptoms. This pattern was also seen in those with elevated PTSD symptoms compared to those with minimal/no symptoms. Prolonged QTc interval has previously been associated with acute mental stress and post-ACS depression in females [30, 31]. QT interval has also been indicated as an electrocardiographic feature that could predict comorbid anxiety and depression [32], however, we did not find this association to be significant in our analysis. A prior study among older adults linked the use of Tricyclic antidepressants and Citalopram to prolonged QT interval [33], while other antidepressant classes like Selective Serotonin Reuptake Inhibitors and Serotonin Norepinephrine Reuptake Inhibitors did not appear to be associated with it. Use of the associated medications among our participants could potentially confound the relationship between TTS and prolonged QT. In the future, it would be helpful to consider the long-term impact of prolonged QTc on TTS recurrence, morbidity, and mortality while controlling for the uptake of these medications.

### Limitations

While it is beneficial to have adjudicated non-TTS participants as a “disease” comparison group due to similar characteristics, this approach limited our reference sample size because the registry is designed to enroll individuals with actual TTS diagnoses. Additionally, the time lapse between TTS events and psychosocial assessments limits our ability to infer causality between psychosocial factors and TTS. However, our use of detailed self-reported scales rather than chart review or claims data provides a deeper insight into the burden of adverse psychosocial characteristics in our registry population with confirmed TTS. Retrospective self-report of the trigger type could be impacted by recall bias, resulting in misclassification of the trigger type. We mitigated this by identifying documented trigger types from the medical records and reporting them in addition to the self-reported trigger classification. Our findings generate hypotheses for future TTS research, as well as highlight differences in comparison with those confirmed to be non-TTS.

## Conclusions

Psychosocial characteristics including PTSD, depression, and stress are common among women with TTS, with age at the time of TTS event a potentially important moderator in this relationship. Depressive symptoms were associated with prolonged QTc in TTS, although it is unclear how this impacts long-term outcomes. We did not find Trait-anxiety or early childhood trauma to be associated with TTS in our cohort. This paper adds to the existing body of knowledge characterizing psychosocial features of individuals with TTS. It also offers new knowledge about important psychosocial factors that may contribute to worse clinical presentations or disease course during a TTS event. Future research is needed to further understand the pathways by which psychosocial factors impact TTS incidence and morbidity.

## Electronic supplementary material

Below is the link to the electronic supplementary material.

Supplementary Material 1

## Data Availability

The data underlying this article will be shared on reasonable request to the corresponding author.

## Declarations

### Availability for data and materials

The data underlying this article will be shared on reasonable request to the corresponding author.

## References

[1] Akashi YJ, Nef HM, Lyon AR. Epidemiology and pathophysiology of Takotsubo syndrome. Nat Reviews Cardiol. 2015;12(7):387–97. 10.1038/nrcardio.2015.39 25855605

[2] Deshmukh A, Kumar G, Pant S, Rihal C, Murugiah K, Mehta JL. Prevalence of Takotsubo cardiomyopathy in the united States. Am Heart J. 2012;164(1):66–. 22795284 10.1016/j.ahj.2012.03.020

[3] Suzuki H, Yasuda S, Shimokawa H. Brain–heart connection in Takotsubo syndrome before onset. Eur Heart J. 2021;42(19):1909–11. 33532845 10.1093/eurheartj/ehab026

[4] Wang X, Pei J, Hu X. The Brain-Heart connection in Takotsubo syndrome: the central nervous system, sympathetic nervous system, and catecholamine overload. Cardiol Res Pract. 2020;2020:1–5. 10.1155/2020/4150291 PMC7085406 32211202

[5] Salmoirago-Blotcher E, Rosman L, Wittstein IS, Dunsiger S, Swales HH, Aurigemma GP, Ockene IS. Psychiatric history, post-discharge distress, and personality characteristics among incident female cases of Takotsubo cardiomyopathy: A case-control study. Heart Lung. 2016;45(6):503–9. 27553636 10.1016/j.hrtlng.2016.07.008

[6] Oliveri F, Goud HK, Mohammed L, Mehkari Z, Javed M, Althwanay A, et al. Role of depression and anxiety disorders in Takotsubo syndrome. The Psychiatric Side of Broken Heart. Cureus; 2020. 10.7759/cureus.10400 PMC7489571 32944484

[7] Obrutu O, Maughan J, Tjoe B, Herscovici R, Moy P, Rojas N et al. Smidt heart Institute Takotsubo Registry – Study design and baseline characteristics. Am Heart J Plus. 2022;13. 10.1016/j.ahjo.2022.100086 PMC10978169 38560083

[8] Ghadri J-R, Wittstein IS, Prasad A, Sharkey S, Dote K, Akashi YJ, et al. International expert consensus document on Takotsubo syndrome (Part I): clinical characteristics, diagnostic criteria, and pathophysiology. Eur Heart J. 2018;39(22):2032–46. 29850871 10.1093/eurheartj/ehy076 PMC5991216

[9] Goetzmann L, Olliges E, Ruettner B, Meissner K, Ladwig K-H, Möller C, et al. Adverse childhood experiences and the structure of personality in patients with Takotsubo syndrome versus myocardial infarction. Heart Mind. 2020;4(1):12–20.

[10] Goh ACH, Wong S, Zaroff JG, Shafaee N, Lundstrom RJ. Comparing anxiety and depression in patients with Takotsubo stress cardiomyopathy to those with acute coronary syndrome. J Cardiopulm Rehabil. 2016;36(2):106–11. 10.1097/HCR.0000000000000152 26468629

[11] Cui KX, Sui P, Zang XH, Sun YY, Liu XH. Development and validation of a risk prediction model for post-traumatic stress disorder symptoms in patients with acute myocardial infarction in China. Ann Palliat Med. 2022;11(9):2897–905. 36217618 10.21037/apm-22-881

[12] Miao X, Chen YL, Qiu XX, Wang RH. Construction and validation of a nomogram predicting depression risk in patients with acute coronary syndrome undergoing coronary stenting: A prospective cohort study. J Cardiovasc Dev Dis. 2023;10(9). 10.3390/jcdd10090385 PMC10532347 37754813

[13] Del Pace S, Parodi G, Bellandi B, Zampini L, Venditti F, Ardito M, et al. Anxiety trait in patients with stress-induced cardiomyopathy: a case-control study. Clin Res Cardiol. 2011;100(6):523–9. 21221609 10.1007/s00392-010-0276-x

[14] Smeijers L, Szabó BM, Kop WJ. Psychological distress and personality factors in Takotsubo cardiomyopathy. Neth Heart J. 2016;24(9):530–7. 27401603 10.1007/s12471-016-0861-3 PMC5005193

[15] Lehavot K, Goldberg SB, Chen JA, Katon JG, Glass JE, Fortney JC, et al. Do trauma type, stressful life events, and social support explain women veterans’ high prevalence of PTSD? Soc Psychiatry Psychiatr Epidemiol. 2018;53(9):943–53. 29936598 10.1007/s00127-018-1550-x PMC6521967

[16] Kessler RC, Sonnega A, Bromet E, Hughes M, Nelson CB. Posttraumatic stress disorder in the National comorbidity survey. Arch Gen Psychiatry. 1995;52(12):1048–60. 7492257 10.1001/archpsyc.1995.03950240066012

[17] Magruder K, Serpi T, Kimerling R, Kilbourne AM, Collins JF, Cypel Y, et al. Prevalence of posttraumatic stress disorder in Vietnam-Era women veterans: the health of Vietnam-Era women’s study (HealthVIEWS). JAMA Psychiatry. 2015;72(11):1127–34. 26445103 10.1001/jamapsychiatry.2015.1786 PMC7529477

[18] Lehavot K, Katon JG, Chen JA, Fortney JC, Simpson TL. Post-traumatic stress disorder by gender and veteran status. Am J Prev Med. 2018;54(1):e1–9. 29254558 10.1016/j.amepre.2017.09.008 PMC7217324

[19] Lee B, Wang Y, Carlson SA, Greenlund KJ, Lu H, Liu Y, et al. National, State-Level, and County-Level prevalence estimates of adults aged = 18 years Self-Reporting a lifetime diagnosis of Depression-United states, 2020. Mmwr-Morbid Mortal W. 2023;72(24):644–50. 10.15585/mmwr.mm7224a1 PMC10328468 37318995

[20] Weihs V, Pogran E, Kunschitz E, Weihs W, Prinz E, Eichenberg C, et al. Psychocardiological assessment in the acute phase of the Takotsubo syndrome. Wiener Klinische Wochenschrift. 2022;134(7–8):269–75. 34671830 10.1007/s00508-021-01957-1 PMC9023402

[21] Christensen TE, Bang LE, Holmvang L, Hasbak P, Kjær A, Bech P, Østergaard SD. Neuroticism, depression and anxiety in Takotsubo cardiomyopathy. BMC Cardiovasc Disord. 2016;16(1). 10.1186/s12872-016-0277-4 PMC4888627 27246461

[22] Galli F, Bursi F, Carugo S. Traumatic events, personality and psychopathology in Takotsubo syndrome: A systematic review. Front Psychol. 2019;10. 10.3389/fpsyg.2019.02742 PMC6914859 31920800

[23] Sundelin R, Bergsten C, Tornvall P, Lyngå P. Self-rated stress and experience in patients with Takotsubo syndrome: a mixed methods study. Eur J Cardiovasc Nurs. 2020;19(8):740–7. 32491953 10.1177/1474515120919387 PMC7817986

[24] Swedo E, Aslam M, Dahlberg L, Holditch Niolon P, Guinn A, Simon T, Mercy J. Prevalence of adverse childhood experiences among U.S. Adults — Behavioral risk factor surveillance system, 2011–2020. MMWR Morb Mortal Wkly Rep 2023. 2023;72(26):707–15. 10.15585/mmwr.mm7226a2 PMC10328489 37384554

[25] Vazirani R, Rodriguez-Gonzalez M, Castellano-Martinez A, Andres M, Uribarri A, Corbi-Pascual M, et al. Pediatric Takotsubo cardiomyopathy: A review and insights from a National multicentric registry. Heart Fail Rev. 2024;29(4):739–50. 38483658 10.1007/s10741-024-10394-x

[26] Nunez-Gil IJ, Santoro F, Vazirani R, Novo G, Blanco-Ponce E, Arcari L, et al. Smoking influence in Takotsubo syndrome: insights from an international cohort. Front Cardiovasc Med. 2023;10:1282018. 38054096 10.3389/fcvm.2023.1282018 PMC10694470

[27] Lu X, Li P, Teng C, Cai P, Jin L, Li C, et al. Prognostic factors of Takotsubo cardiomyopathy: a systematic review. ESC Heart Fail. 2021;8(5):3663–89. 34374223 10.1002/ehf2.13531 PMC8497208

[28] Isogai T, Okada A, Morita K, Michihata N, Makito K, Matsui H, et al. Body mass index and outcomes in patients with Takotsubo syndrome: A nationwide retrospective cohort study. Cardiology. 2024;149(4):314–24. 38387447 10.1159/000537971 PMC11309069

[29] Zalewska-Adamiec M, Malyszko J, Bachorzewska-Gajewska H, Tomaszuk-Kazberuk A, Dobrzycki SJ. Takotsubo syndrome - fatal prognosis of patients with low body mass index in 5-year follow-up. Arch Med Sci. 2020;16(2):282–8. 32190137 10.5114/aoms.2019.87082 PMC7069448

[30] Whang W, Julien HM, Higginbotham L, Soto AV, Broodie N, Bigger JT, et al. Women, but not men, have prolonged QT interval if depressed after an acute coronary syndrome. Europace. 2012;14(2):267–71. 21798879 10.1093/europace/eur246 PMC3262404

[31] Andrássy G, Szabo A, Ferencz G, Trummer Z, Simon E, Tahy Á. Mental stress May induce QT-Interval prolongation and T‐Wave Notching. Ann Noninvasive Electrocardiol. 2007;12(3):251–9. 17617071 10.1111/j.1542-474X.2007.00169.x PMC6932412

[32] Tang MC, Xi JZ, Fan XW. QT interval is correlated with and can predict the comorbidity of depression and anxiety: A cross-sectional study on outpatients with first-episode depression. Front Cardiovasc Med. 2022;9. 10.3389/fcvm.2022.915539 PMC9559700 36247470

[33] Rochester MP, Kane AM, Linnebur SA, Fixen DR. Evaluating the risk of QTc prolongation associated with antidepressant use in older adults: a review of the evidence. Ther Adv Drug Saf. 2018;9(6):297–308. 29854391 10.1177/2042098618772979 PMC5971403

