# Supplementary material for: Psychosocial and clinical characteristics in Takotsubo syndrome
Source: Biol Sex Differ. 2025 Jun 16;16:42. doi: 10.1186/s13293-025-00729-0 (PMC12168290; doi:10.1186/s13293-025-00729-0)
Supplement: Supplementary file 1 — Supplementary Material 1 [file 13293_2025_729_MOESM1_ESM.docx]

**International Takotsubo Diagnostic Criteria (InterTAK Diagnostic Criteria)**

1. **Transient**⁽ᵃ⁾ left ventricular dysfunction (hypokinesia, akinesia or dyskinesia) presenting as apical ballooning or midventricular, basal or focal wall motion abnormalities. Right ventricular involvement can be present. Besides these regional wall motion patterns, transitions between all types can exist. The regional wall motion abnormality usually extends beyond a single epicardial vascular distribution; however, rare cases can exist where the regional wall motion abnormality is present in the subtended myocardial territory of a single coronary artery (focal TTS).⁽ᵇ⁾
2. An emotional, physical or combined trigger can precede the Takotsubo syndrome event, but this is not obligatory.
3. Neurologic disorders (e.g., subarachnoid hemorrhage, stroke/transient ischemic attack, or seizures) as well as pheochromocytoma may serve as triggers for takotsubo syndrome.
4. New ECG abnormalities are present (ST-segment elevation, ST-segment depression, T-wave inversion, and QTc prolongation); however, rare cases exist without any ECG changes.
5. Levels of cardiac biomarkers (troponin and creatine kinase) are moderately elevated in most cases; significant elevation of brain natriuretic peptide is common.
6. Significant coronary artery disease is not a contradiction in takotsubo syndrome.
7. Patients have no evidence of infectious myocarditis.
8. Post-menopausal women are predominantly affected.

⁽ᵃ⁾ Wall motion abnormalities may remain for a prolonged period or documentation of recovery may not be possible. For example, death before evidence of recovery is captured.
⁽ᵇ⁾ Cardiac magnetic resonance imaging is recommended to exclude infectious myocarditis and diagnosis confirmation of takotsubo syndrome.

Source: J.-R. Ghadri, I.S. Wittstein, A. Prasad, et al. International expert consensus document on takotsubo syndrome (Part I): clinical characteristics, diagnostic criteria, and pathophysiology. Eur. Heart J., 39 (22) (2018), pp. 2032-2046, 10.1093/eurheartj/ehy076
